# Pitfalls in Geriatric Prescribing: Antidepressants and Extreme Sedation

**DOI:** 10.1155/2019/4290207

**Published:** 2019-05-13

**Authors:** Hirofumi Namiki

**Affiliations:** Tokachi- Ikeda Community Medical Center, Japan Association for Development of Community Medicine, Ikeda, 0830022 Hokkaido, Japan

## Abstract

In this study, a case is presented in which initiation of an antidepressant drug was associated with an episode of extreme sedation. This case provides an opportunity to highlight possible pitfalls in geriatric prescribing.

## 1. Introduction

Coupland et al. [1] have previously reported the risk of adverse effects secondary to the use of antidepressants in the elderly. Antidepressants are commonly prescribed for a range of therapeutic indications, such as treatment of depression and management of neuropathic pain [2]. These uses are associated with several well-recognized adverse effects, including sexual dysfunction, weight change, bleeding, cardiovascular effects, and anticholinergic effects [3]. However, another significant adverse effect of antidepressant drugs is extreme sedation.

## 2. Case Presentation

A 92-year-old man was admitted to hospital with a general decline in functional status. A comprehensive geriatric assessment revealed low mood without evidence of cognitive impairment, and a diagnosis of depression was made. The patient was prescribed mirtazapine 7.5 mg nocte per oral, and the following day, he became increasingly unstable. Two days later, the patient was found unresponsive in bed.

There was no history of head trauma, and no seizure activity was observed. Vital signs were unremarkable, while respiratory, cardiovascular, and abdominal examinations were normal. However, neurological examination revealed diminished mental status. The patient did respond to a deep, painful stimulus but his eyes remained closed, and there was no verbal response. The patient had brisk deep tendon reflexes and showed plantar reflexes of the extensor. Blood tests (including those for urea, electrolytes, glucose, calcium, magnesium, Vitamin B12 and folate levels, C-reactive protein, thyroid function, and full blood count) were normal. An electrocardiogram displayed normal sinus rhythm, and an emergency magnetic resonance brain scan demonstrated no evidence of acute intracranial pathology.

The patient's level of consciousness gradually returned to normal after 4 hours. The antidepressant-induced sedation was suspected of being the cause, and mirtazapine was immediately stopped. There was no subjective improvement in mood after withdrawal of mirtazapine. Three days later, the patient was re-prescribed on mirtazapine 3.75 mg nocte. Though he appeared expressionless, he gradually became more responsive after a week. Following the reduction of mirtazapine dose, the patient did not experience any more episodes of extreme sedation during a 2-month follow-up.

## 3. Discussion

This case is a teaching example of antidepressant-induced extreme sedation in a geriatric prescription for depression. Depression is the most common mental health disorder with life-threatening consequences and affects up to 13.3% of the elderly population [4]. Beekman et al. [5] reported that disability is an essential determinant in the prevalence of depression in the elderly, with inpatients with long-term conditions and those living in adverse socioeconomic circumstances having a higher prevalence than people living in the community and those of average socioeconomic status. In this case, following the withdrawal of the antidepressant, a smaller dose of antidepressant was reintroduced to avoid extreme sedation. The patient experienced an additional, yet reduced sedative episode following a smaller dose of antidepressant. This episode could conclusively prove that the antidepressant had produced the extreme sedation. The sedative effect of antidepressants has been rarely discussed. This case provides an opportunity to highlight the importance of depression in this population, as well as potential behavioral mechanisms associated with antidepressants, and more generally, appropriate prescribing in the elderly.

According to the NHS Grampian Guidance for Initiating Antidepressants, which was developed according to guidance by the National Institute for Health and Care Excellence, mirtazapine and sertraline are both first-line treatments for depression in older people [6, 7]. Mirtazapine is classified as atypical antidepressant and described as a noradrenergic and specific serotonergic antidepressant (NaSSA). Mirtazapine is specifically a potent antagonist or inverse agonist of the alpha2-adrenergic receptor, the serotonin 5-HT2 receptor, 5-HT3 receptor, H1 histamine receptor, a moderate peripheral alpha-adrenergic receptor, and muscarinic receptor antagonist. Moreover, antimuscarinic action in mirtazapine could lead to sedation during initial treatment. Indeed, mirtazapine is usually given at night due to its sedating effect [8, 9].

Sertraline is another antidepressant that can also cause drowsiness [10]. On acute use, sertraline can selectively block serotonin reuptake and can increase serotonin stimulation of somatodendritic 5HT1 but has only weak effects on norepinephrine and neuronal dopamine uptake [11]. However, there is evidence that sertraline has a benefit over other classes of antidepressants in terms of safety or tolerability for the acute phase of severe depression treatment [12].

Prescribing for older patients presents unique challenges because the elderly are often more sensitive to drugs and therefore are at increased risk of experiencing adverse effects. This is due to the pharmacokinetic and pharmacodynamic changes that occur with aging, including increased body fat composition, decreased lean mass, decreased P450 enzyme system activity, decreased renal excretion of drugs, and increased sensitivity to the effects of drugs. These factors are especially important when the drug involved has a narrow therapeutic range, which is the case with antidepressants [8, 13].

Furthermore, adverse drug reactions commonly lead to hospital admission in the elderly [14–16]. According to Beijer and de Blaey [17], adverse drug reactions result in four times as many hospitalizations in older adults than in younger adults. The possibility of an adverse drug event should always be considered by clinicians when evaluating an older adult. In general, prescribing in the elderly should follow the pattern of “start low, go slow” to reduce the possibility of an adverse drug event. Moreover, polypharmacy is often observed in older people due to the presence of multiple comorbidities [8, 13]. Therefore, it is crucial that drugs be regularly reviewed and adverse drug effects actively considered, such that medications that do not provide significant clinical benefit are discontinued.

Prescription of potentially inappropriate and excessive medications in older people is common. A review article by Gallagher et al. [18] reported that inappropriate and excessive medications are often prescribed, ranging from 12% of community-dwelling older people to 40% of nursing home residents in the USA and Europe. Given this high prevalence, the review recommends the use of pharmacogenetic decision support tools, which consider evidence-based prescription in the context of the clinician's practice, with consideration of diverse patient needs within each country's national formulary [19].

Additional discussion would be needed in elderly patients as follows. Many patients with depression who do not respond adequately to standard treatment with pharmacotherapy and psychotherapy are candidates for noninvasive neuromodulation procedures, including transcranial magnetic stimulation (TMS) and electroconvulsive therapy (ECT) [20, 21]. Patients, especially the elderly, may prefer TMS because it is better tolerated; TMS does not require general anesthesia and induction of seizures. TMS, including deep TMS approved by the FDA, is a noninvasive, clinically available treatment with demonstrated efficacy [22].

Despite the effectiveness of pharmacological treatments in depression, symptom remission has been achieved in fewer than 40% of elderly patients of depression with cognitive impairment, with or without dementia [23]. Therefore, effective psychosocial interventions for this patient population are needed. Most of the psychosocial interventions for geriatric depression are designed for young-old (average age, 65 to 70 years) and cognitively intact outpatients who could follow adequate treatment plans [24]. To effectively treat depressed elderly patients with comorbid cognitive impairment and disability, psychosocial interventions need to be comprehensively modified. With these modifications, psychosocial interventions could provide relief to many groups of elderly patients and synchronize to the elderly people's abilities, preferences, and needs aiming to prevent the onset of depression.
